# Decline of protein structure rigidity with interatomic distance

**DOI:** 10.1186/s12859-021-04393-0

**Published:** 2021-09-28

**Authors:** Oliviero Carugo

**Affiliations:** 1 Department of Chemistry, University of Pavia, Pavia, Italy; 2 Department of Structural and Computational Biology, University of Vienna, Campus Vienna Biocenter 5, 1030 Vienna, Austria

**Keywords:** Anisotropy, B-factor, Crystallography, Hirshfeld test, Protein rigidity, Protein structure

## Abstract

**Background:**

Protein structural rigidity was analyzed in a non-redundant ensemble of high-resolution protein crystal structures by means of the Hirshfeld test, according to which the components (*uX* and *uY*) of the B-factors of two atoms (*X* and *Y*) along the interatomic direction is related to their degree of rigidity: the atoms may move as a rigid body if *uX* = *uY* and they cannot if *uX* ≠ *uY*.

**Results:**

It was observed that the rigidity degree diminishes if the number of covalent bonds intercalated between the two atoms (*d_seq*) increases, while it is rather independent on the Euclidean distance between the two atoms (*d*): for a given value of *d_seq*, the difference between *uX* and *uY* does not depend on *d*. No additional rigidity decline is observed when *d_seq* ≥  ~ 30 and this upper limit is very modest, close to 0.015 Å.

**Conclusions:**

This suggests that protein flexibility is not fully described by B-factors that capture only partially the wide range of distortions that proteins can afford.

**Supplementary Information:**

The online version contains supplementary material available at 10.1186/s12859-021-04393-0.

## Background

Molecule flexibility is inherent in thermodynamic stability and chemical reactivity [1]. In globular proteins, for example, the residual mobility of solvent exposed side-chains and loops may provide a favorable entropic contribution to the folding free energy [2, 3] and it may tune the thermodynamics of substrate access into active sites—and of course the exit of products [4, 5]—and of binding partner recognition [2, 6].

Studies on protein flexibility have addressed numerous molecular features by means of several methodological approaches. Atomic resolution crystallography allowed the characterization of conformationally disordered atoms [7–10]. Time resolved crystallography provided three-dimensional models of dynamical changes that occur during chemical reactions [11]. Molecular dynamics studies allowed simulations of macromolecular movements in silico [12, 13] and the estimation of thermodynamic state functions [14]. Other computational approaches, like normal mode analysis, have been used to identify the structural distortions of a protein about an equilibrium position [15].

Another source of information about protein flexibility is provided by the atomic displacement parameters—usually referred to as B-factor (B)—that monitor the positional displacements of the atoms around their equilibrium positions [16, 17]. B-factors have been used in numerous studies to analyze protein dynamics [18, 19]. Although they are, in general, determined and refined isotropically, they are particularly informative in atomic resolution protein crystal structures, when they can be refined anisotropically due the abundance of experimental diffraction data [20].

Here a new and insofar unexplored aspect is considered: how does flexibility decrease when the separation between atoms increases. It can be expected that flexibility is minimal for covalently bound atoms and, more in general, for atoms close to each other, since close interatomic contacts tend to be rigid [21]—this is reflected in molecular modelling by the attribute of hardness given to covalent bond and angles [22]. On the contrary, distant atoms are not expected to behave as a rigid body and their movements can be, to some extent at least, uncorrelated.

Flexibility degree can be monitored by means of the Hirshfeld test [23], which employs the B-factor: for a rigid contact between two atoms X and Y, the components along the interatomic direction of the B-factors of the two atoms (u_X_ and u_Y_) must be identical. This means that their difference (Delta-u) must be equal to zero Å:

1 $$Delta - u = \left| {u_{X} - u_{Y} } \right| = 0 {\AA}$$

On the contrary, Delta-u far from zero Å is expected for atoms that do not behave as a rigid body and have displacements and dispersions around their average locations independent of each other.

Atom pair separation is defined in two different ways. On the one hand, it is the Euclidean distance (d) between the atoms and, on the other, it is the number of covalent bonds intercalated between the atoms (covalent separation, d_seq).

It is observed that Delta-u values increase if d or d_seq increase. However, the dependence of Delta-u on d is likely to be due to the fact that d is proportional to d_seq. In fact, for a given value of d_seq, Delta-u does not depend on d.

Moreover, it is observed that Delta-u tends to rich its maximal value at d_seq ≈ 30 and to be nearly constant for d_seq > 30. This maximal value is considerably smaller if the Delta-u values are computed with anisotropic B-factors than with isotropic B-factors, suggesting that the isotropic B-factors overestimate protein flexibility.

The maximal Delta-u values are however very modest, close to 0.015 Å, indicating that B-factors are rather unrelated, on average, to the stereochemical rearrangements, which are known to confer high flexibility to proteins, for example for exchanging buried water molecules with the external solvent.

## Results

Delta-u values, Euclidean distances and covalent separations were computed for 6,794,404 pairs of atoms in 30 crystal structures, with covalent separation up to 50.

**Fig. 1:**
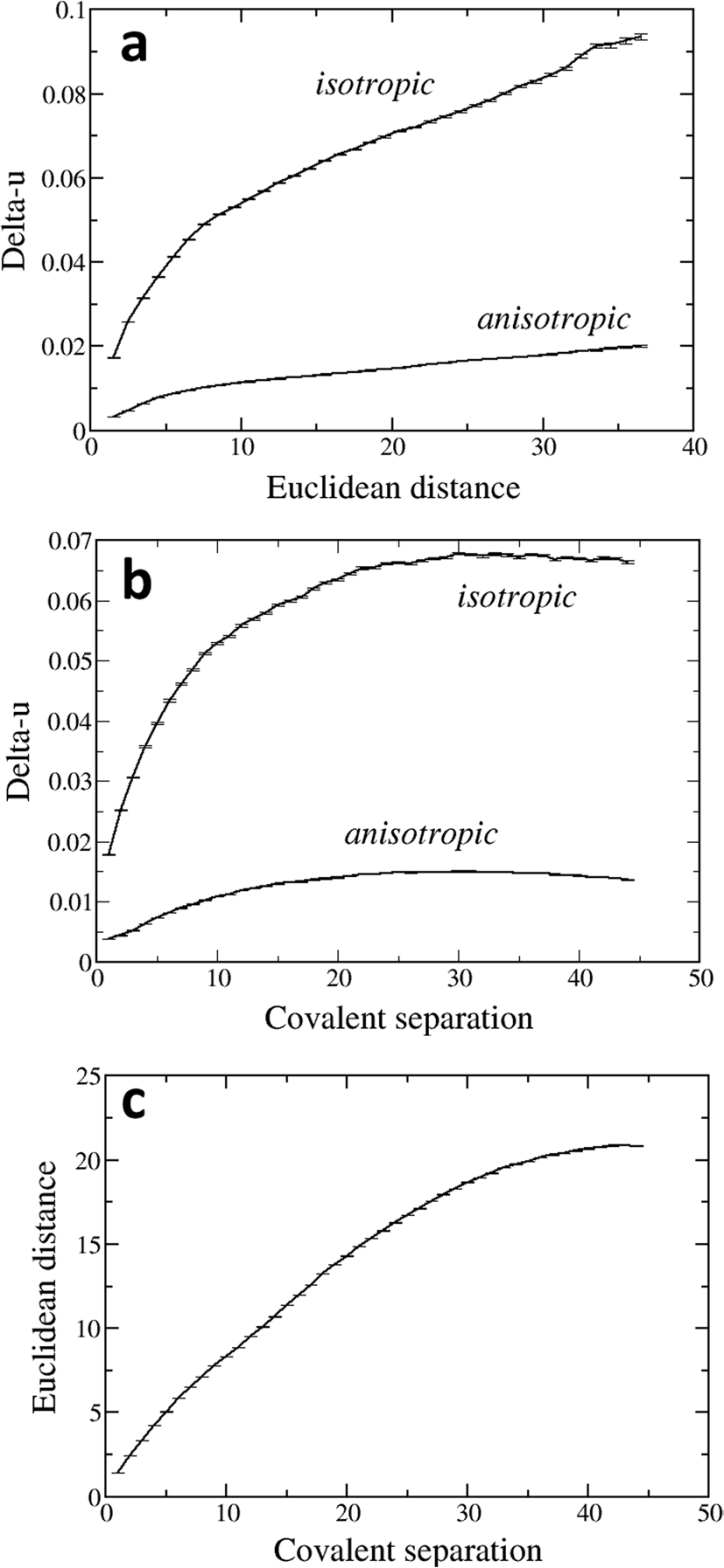
Relationships between isotropic and anisotropic Delta-u (Å) and Euclidean distance (Å; **a**) and covalent separation (**b**), and relationship between Euclidean distance (Å) and covalent separation (**c**; error bars show the estimated standard deviations)

The relationships between Delta-u and Euclidean distance or covalent separation are shown in Fig. 1. Several, interesting observations can be done.

First, the flexibility of atom pairs is clearly overestimated by isotropic Delta-u. This is not unexpected, since anisotropically refined B-factors represent better the positional scatter of the atoms. It is however surprising that the difference between isotropic and anisotropic Delta-u is so large: for atoms 30–35 Å apart, the isotropic Delta-u (ca. 0.08–0.09 Å) is about 4 times larger than its anisotropic counterpart (ca. 0.02 Å); and for atoms separated by 30 covalent bonds it (ca. 0.065 Å) is about 4 times larger than the anisotropic Delta-u (ca. 0.015 Å).

Second, a difference between Euclidean distances and covalent separation appears too. The Delta-us, both isotropic and anisotropic, tend to increase with Euclidean distance and the increase is rather linear for Euclidean distances larger than 10 Å (Fig. 1a). On the contrary, they do not increase monotonically when the covalent bond separation increases (Fig. 1b): in this case, the Delta-us reach a plateau when the covalent separation overtakes 25–30 covalent bonds. The different relationships between Delta-u and Euclidean distances, one the one hand, and covalent separation, on the other, might reflect the fact that the relationship between Euclidean distance and covalent separation is not linear (Fig. 1c).

Third, and this is not surprising, the rigidity of atom pairs decreases when the distance—either the Euclidean or the covalent separation—between them increases. It is obviously expected that covalently bound atoms present a rigid body behavior while distant atoms may present a considerable flexibility, limited by the natural compactness of the globular proteins.

Detailed data on the relationships of anisotropic Delta-u with Euclidean distance and covalent separation are shown in Table 1 (an analogous table is not reported here for isotropic Delta-u, since the same trends are observed). It appears that the dependence of Delta-u on the two distances is different. Given a certain covalent separation, Delta-u is substantially independent of the Euclidean distance. For example, at short covalent separations equal to 6, the Delta-u oscillates slightly between 0.007 and 0.008 Å if the Euclidean distance goes from 3.5 to 7.5 Å; and at longer covalent separation equal to 20, the Delta-u oscillates only between 0.010 and 0.013 Å if the Euclidean distance goes from 3.5 to 21.5 Å.

**Table 1:** Anisotropic Delta-u values (× 1000; Å) as a function of the Euclidean distance (horizontal, Å) and of the covalent separation (vertical)

	1.5	2.5	3.5	4.5	5.5	6.5	7.5	8.5	9.5	10.5	11.5	12.5	13.5	14.5	15.5	16.5	17.5	18.5	19.5	20.5	21.5	22.5	23.5	24.5	25.5	26.5	27.5	28.5	29.5	30.5
1	3																													
2		4	3																											
3		5	5	5																										
4		7	6	6	7																									
5		8	6	7	7	7																								
6			7	8	8	8	7																							
7			9	8	9	10	9	8																						
8		9	10	9	9	10	10	9	9																					
9			9	10	10	10	10	10	10	9																				
10			9	10	10	10	10	11	11	10	8																			
11		11	9	10	10	10	10	10	11	10	9	8	10																	
12			10	10	11	12	11	11	11	11	11	10	8	11																
13			11	10	11	11	11	11	11	11	11	10	10	9	9															
14		10	11	10	10	11	12	12	11	12	11	11	11	10	9	8														
15		9	11	10	10	11	11	11	11	11	11	11	10	10	10	9	8													
16			10	10	10	11	11	12	11	11	11	11	11	10	10	9	9	10												
17			11	11	10	11	11	12	12	12	12	11	11	11	11	10	10	9	9											
18			10	11	11	11	12	12	12	12	12	12	11	12	11	11	10	10	10	9										
19			12	12	11	12	12	13	13	13	13	13	12	12	12	12	11	11	10	10	10									
20			13	11	11	12	12	13	13	13	13	13	12	12	12	12	11	11	11	11	10	11								
21			14	13	13	13	13	14	14	14	14	14	13	13	13	13	13	13	12	12	11	11	13							
22			14	13	13	13	13	14	14	13	14	13	13	13	13	13	13	13	12	12	12	12	12	13						
23			15	13	13	14	14	14	14	14	14	14	14	13	13	13	13	13	13	12	12	12	12	12	12					
24			17	14	15	15	15	15	15	15	15	15	15	15	14	14	14	14	15	14	13	13	13	12	12	14				
25			19	16	17	17	17	17	16	17	17	17	16	17	16	15	15	15	15	16	14	14	13	15	14	15	15			
26			18	16	19	18	18	17	17	17	16	16	16	16	16	16	16	16	16	16	16	15	14	13	14	14	13	17		
27			23	20	22	21	20	19	18	18	18	17	17	17	17	17	17	17	18	17	17	17	15	15	15	15	16	15	18	
28			23	24	21	23	21	20	18	18	17	18	17	17	17	17	17	17	17	17	18	17	17	16	15	15	17	18	16	20
29			27	22	22	22	20	19	18	18	17	17	17	17	16	16	17	16	17	16	16	17	17	16	15	14	15	16	17	15
30			27	21	23	21	19	17	18	18	17	17	17	18	17	16	16	17	17	16	17	17	17	16	15	15	15	15	16	17
31			28	23	22	21	19	18	18	17	18	18	18	18	18	17	17	17	16	17	17	17	17	18	17	16	16	16	20	19
32			25	23	22	20	18	18	17	17	18	17	17	17	17	17	17	17	16	17	16	16	17	17	17	17	16	16	18	20
33			18	24	22	22	21	19	19	18	18	18	17	18	19	19	18	18	17	18	16	16	16	16	16	17	17	16	18	18
34			20	21	22	21	21	19	19	18	18	18	17	17	17	18	17	17	17	17	17	16	16	16	16	16	16	16	15	16
35			14	25	24	22	20	20	19	19	18	18	18	18	17	18	18	18	18	18	18	18	17	17	17	18	19	18	19	19
36			17	21	20	19	20	20	20	20	20	19	19	18	18	18	19	19	19	19	19	19	18	18	17	17	18	18	17	17
37			15	20	18	19	19	19	21	20	19	20	19	19	18	18	18	19	19	19	19	18	19	18	19	19	20	19	19	17
38			18	21	20	20	19	19	19	19	19	19	19	18	18	18	19	18	18	18	18	18	18	18	18	19	19	20	20	19
39			21	19	19	19	18	18	19	20	19	19	19	18	18	19	18	19	18	18	18	18	18	18	18	19	19	20	19	20
40			23	19	18	18	19	18	19	19	19	18	19	19	19	19	19	18	19	18	18	18	19	18	18	18	18	19	18	19

Delta-u values are not provided if there are less than 50 observations. The estimated standard errors, not reported for simplicity, oscillates from 1 to 2 (× 1000; Å)

This suggests that the rigidity decline is strongly connected to the covalent separation and its dependence on Euclidean distance is simply a consequence of the fact that Euclidean distance is somehow related to covalent separation.

To prove that these trends are significant, despite this is an observational study based on data available at the Protein Data Bank, the 30 crystal structures examined in this manuscript were randomly divided into three, equally populated groups. The relationships between Delta-u and covalent separation determined in the three subsets (Additional file 1: Figure S1) are very similar. This strongly supports the validity of the trends described above, though any deeper interpretation is hindered, at least in part, by the fact that the estimated errors of the B-factors deposited in the Protein Data Bank are unknown—as well as the estimated errors on the atomic coordinates.

## Discussion

The level of rigidity of protein structures can be estimated by the variable Delta-u (see Eqs. 3 and 5), the value of which is expected to be equal to zero for atom pairs that behave as a rigid body. Obviously, this occurs when the two atoms are covalently bound and very close to each other, while Delta-u values larger than zero are expected for atoms very distant from each other.

Actually, Delta-u values are observed to increase progressively if the interatomic distance increases, either when the interatomic distance is the Euclidean distance (Fig. 1a) or the number of covalent bonds intercalated between the two atoms (Fig. 1b).

However, the dependence of Delta-u on Euclidean distance is probably a consequence of the fact Euclidean distance depends on covalent separation (Fig. 1c). In fact, as it is shown in Table 1, Delta-u is rather independent of Euclidean distance at each value of covalent separation—each line in the table. This suggests that protein rigidity is largely due to its covalent structure and less to non-bonding interactions amongst moieties far from each other along the sequence. Certainly, covalent connections between atoms separated by numerous backbone covalent bonds can exist, for example disulfide bonds or contacts mediated by metal cations, and they contribute to confer some rigidity to the protein. However, most of the contacts between atoms separated by numerous backbone covalent bonds involve van der Waals interactions, which apparently do not confer much rigidity to the protein despite the high protein packing efficiency. Further studies are nevertheless necessary to reach a deeper understanding of this phenomenon.

At large distances, the Delta-u approaches the upper value close to 0.06–0.07 Å, computed with isotropic B-factors (Eq. 5), which is considerably larger than the upper value close to 0.015–0.02 Å, computed with anisotropic B-factors (Eq. 3). This clearly indicates that protein flexibility is enormously overestimates by isotropic B-factors.

These Delta-u values are nevertheless considerably small. This is quite surprising since globular proteins are known to be quite flexible, even if they are compact. For example, water molecules buried into the protein core easily exchange with bulk solvent by opening transient channels that allow the entrance/exit of water [24, 25]. Also, aromatic side-chains are known to flip, with 180° rotation, with high flip rates [26].

All these processes require atomic displacements that are considerably larger than the upper Delta-u limits observed in the present communication.

It can be hypothesized that these considerable local deformations, which allow water molecules to enter in and exit from the protein core and that allow aromatic ring flipping, are due to conformational transitions that do not depend on progressive rigidity loss. For example, it is possible to imagine side-chains that pass from a stable, rotameric conformation to another one, both being relatively rigid; or it is possible to imagine a rearrangement of the hydrogen bond network, with stable hydrogen bonds being broken and being replaced by equally stable, new hydrogen bonds. The classic hinge motions of rigid structural moieties might also disconnected from B-factors [27].

Therefore, even if B-factors are known since long time to monitor conformational strain [28], which larger B-factor being associated with dihedral angles far from their stable values, it is possible to hypothesize that B-factors cannot provide information about transitions from a stable structure to a similarly stable but different conformation, which are often referred to as conformational sub-states [29–31].

A metaphor for this phenomenon can be an auditorium, all the seats of which are occupied by spectators that can exchange their seats: before and after the exchange, the ensemble of spectators is rather compact and rigid, while a large flexibility is observed when the spectators move from a one seat to another, exchanging their position.

**Fig. 2:**
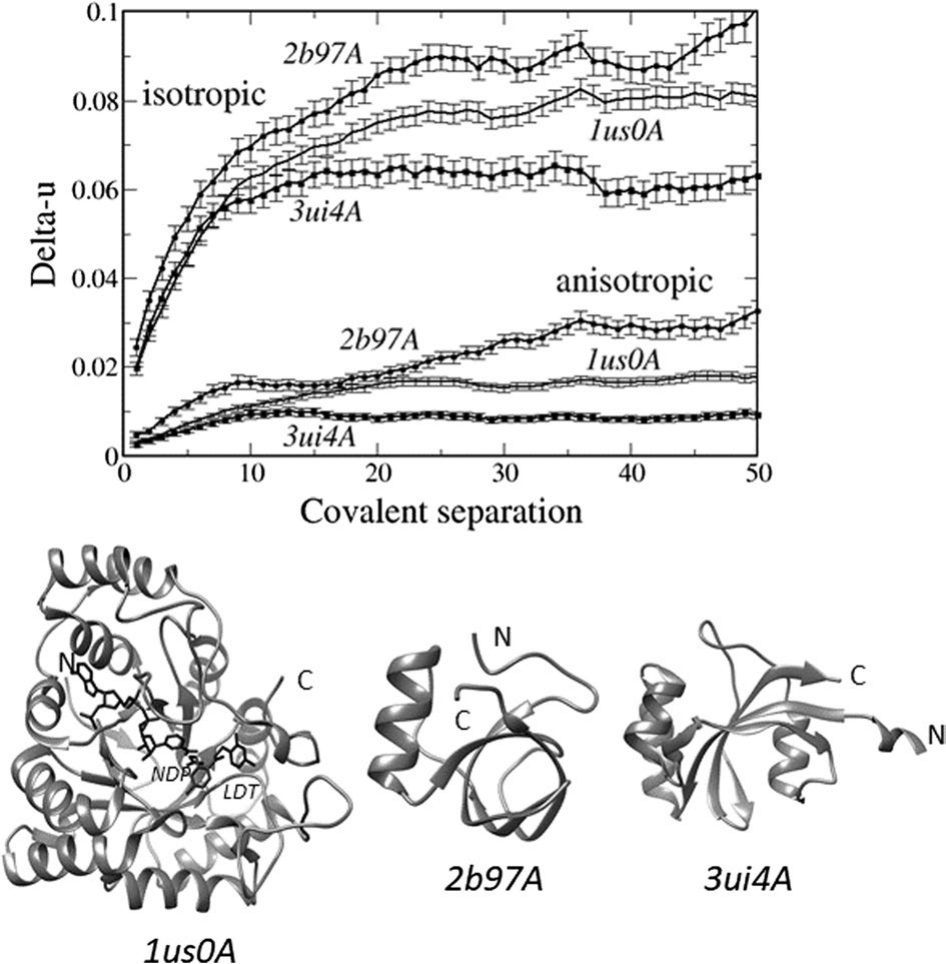
Relationship between isotropic and anisotropic Delta-u and inter-atomic covalent separation for three proteins, chains A of 1us0 (human aldose reductase in complex with NADP (NDP) and an inhibitor (LFT)), chain A of 2b97 (*Trichoderma reesei* hydrophoibin), and chain A of 3ui4 (human parvulin 14)

Interestingly, this trend seems to be independent of protein dimension, type of fold, secondary structure composition or biochemical function. As an example, Fig. 2 shows the relationship between Delta-u and covalent separation for three proteins, two of which are enzymes (human aldose reductase, 1us0, and human parvulin, a small peptidyl-prolyl isomerase, 3ui4) and one of which is not (*Trichoderma reesei* hydrophoibin, a small fungal protein that spontaneously forms amphiphilic monolayers). They adopt different fold types, a TIM-barrel for 1us0, essentially a β-barrel for 2b97, and a α-β-α roll for 3ui4, and one of them, 1us0, is much larger than the others. These proteins show similar trends and there are no enormous differences between them; furthermore, the difference between the two enzymes is comparable to their difference from hydrophoibin, and the largest protein (1us0) is intermediate between the other two.

Crystallographic B-factors are largely unable to monitor transitions amongst conformational sub-states. This has been observed, implicitly, in some previous studies. For example, according to a recent study, protein conformational entropy, defined as the movements of certain groups in proteins, is not monitored quantitatively by crystallographic B-factors [32]. Also, it was observed that crystallographic B-factors underestimate the positional heterogeneity in protein crystals [33].

These observations can be explicated as it follows. Crystal structures show the dominating and most stable protein conformation while alternative sub-states remain undetected, especially at low resolution. Some conformational disorder can be observed and refined experimentally only at high resolution [7–10]. B-factors therefore describe the positional scattering around one conformation and do not reflect the more complex conformational flexibility of proteins. Moreover, B-factors do not monitor only the atomic oscillations around equilibrium positions but depend also on crystal heterogeneity in spaced and time. Crystal structures are in effect representations of the electron density maps of the asymmetric unit, which are the average electron density maps computed (1) on all the asymmetric units present in the crystal and (2) with diffraction data measured over a certain time lapse.

As a consequence, B-factors can be computed quite successfully in—very—small molecule crystals, independently of diffraction data, where B-factors monitor quite effectively atomic fluctuations. The vibrational component of the atomic displacement parameter can be computed with quantum chemistry computations in crystals with very small asymmetric units. For example, density functional theory (DFT)-based methods were used for crystalline l-alanine and crystalline urea [34], and density functional perturbation theory was applied to stishovite and quartz [35]. Recently, B-factors have been computed from ab initio phonon frequencies and displacements for elemental crystals of magnesium, ruthenium, cadmium and silicon [36].

On the contrary, protein crystallographic B-factors are affected by too many non-vibrational components and cannot be predicted by computing the energy of the environment of the atoms by means of quantum chemistry approaches, though it has been shown that protein B-factors are somehow correlated to packing density [37]. At this regard, it is noteworthy that B-factors have also been used to estimate atomic coordinate errors [38, 39], based on the diffraction precision index of Cruickshank [40]. Consequently, they cannot be reproduced reliably in silico, independently of diffraction data.

It must be remembered too that most of protein crystal structure information is being produced at low temperature—100 K—and that a different flexibility might be detected at room temperature or at physiological temperature [41]. However, cryo-crystallography is the predominant form of macromolecular crystallography, given its advantages in reducing radiation damage, especially in modern, high brilliance synchrotron beam lines [42–44].

The above discussion does not imply that crystallographic B-factors are of limited value and disconnected from the physicochemical nature of proteins. For example, information about local flexibility can be extracted from B-factor analyses, for example for protein-DNA complexes [45], cold adaptation of psychrophilic enzymes has been shown to be closely related to B-factors [46, 47], and a procedure called B-Fit has been proposed for increasing the thermostability of enzymes and allows their use in chemistry and biotechnology [19]. More in general, protein regions characterized by large B-factors can be considered to be very mobile, though not necessarily rigid; it clearly appears that protein flexibility is not fully described by B-factors, which capture only partially the wide range of distortions that proteins can afford.

## Conclusions

While covalently bound atoms form a rigid structural unit, this rigidity, monitored through the Hirshfeld Delta-u [23], is progressively lost if the number of covalent bonds intercalated between two atoms increases, until 30 covalent bonds, after which the Delta-u is rather constant, close to 0.065 Å, if the rigidity is estimated with isotropic B-factors, or close to 0.015 Å, if the rigidity is estimated with anisotropic B-factors. On the one hand, this clearly shows how rigidity is underestimated in isotropically refined crystal structures and, on the other hand, both upper Delta-u values are smaller than expected, suggesting that B-factors capture only partially the wide range of distortions that proteins can afford.

## Materials and methods

30 crystal structures were extracted from the Protein Data Bank [48, 49] according to the following criteria: redundancy was reduced to 40% pairwise sequence identity [50, 51] in a set of crystal structures determined at 90–110 K and refined at least at 0.8 Å resolution (Additional file 1: Table S1).

The Delta-u values were computed with anisotropic B-factors (U)

2 $${\mathbf{U}} = \left[ {\begin{array}{*{20}c} {{\rm U}_{11} } & {{\rm U}_{12} } & {{\rm U}_{13} } \\ {{\rm U}_{21} } & {{\rm U}_{22} } & {{\rm U}_{23} } \\ {{\rm U}_{31} } & {{\rm U}_{32} } & {{\rm U}_{33} } \\ \end{array} } \right]$$

according to

3 $$Delta - u = \left| {{\varvec{n}}^{T} {\mathbf{U}}_{{\varvec{X}}} {\varvec{n}} - {\varvec{n}}^{T} {\mathbf{U}}_{{\varvec{Y}}} {\varvec{n}}} \right|$$

where ***n*** is the unit vector from atom X to atom Y. These values are referred to as anisotropic Delta-u, to distinguish them from the isotropic Delta-u, computed with the isotropic B-factor equivalent, defined as

4 $$B = 8\pi^{2} \frac{{{\rm U}_{11} + {\rm U}_{22} + {\rm U}_{33} }}{3},$$

by means of the following expression.

5 $$Delta - u = \left| {u_{X} - u_{Y} } \right| = \left| {\sqrt {\frac{{B_{X} }}{{8\pi^{2} }}} - \sqrt {\frac{{B_{Y} }}{{8\pi^{2} }}} } \right|$$

All computations were performed with locally written software.

## Supplementary Information

**Additional file 1. Table S1:** List of the entries of the Protein Data Bank examined in the present article. **Figure S1**: Relationship between Delta-u and covalent separation in three equally populated subsets of the structures examined in the present communication.

## Data Availability

All data generated or analysed during this study are included in this published article [and its Additional file 1].
