# Supplementary material for: Decline of protein structure rigidity with interatomic distance
Source: BMC Bioinformatics. 2021 Sep 28;22:466. doi: 10.1186/s12859-021-04393-0 (PMC8479892; doi:10.1186/s12859-021-04393-0)
Supplement: Supplementary file 1 — Additional file 1. Table S1: List of the entries of the Protein Data Bank examined in the present article. Figure S1: Relationship between Delta-u and covalent separation in three equally populated subsets of the structures examined in the present communication. [file 12859_2021_4393_MOESM1_ESM.docx]

Decline of protein structure rigidity with interatomic distance

Oliviero Carugo

Department of Chemistry, University of Pavia, Pavia (Italy) and Department of Structural and Computational Biology, University of Vienna, Vienna (Austria). ORCID 0000-0002-2924-9016

# Correspondence to

Oliviero Carugo

Department of Structural and Computational Biology

University of Vienna

Campus Vienna Biocenter 5

1030 Vienna (Austria)

Email: [oliviero.carugo@univie.ac.at](mailto:oliviero.carugo@univie.ac.at)

# Supplementary information

| **Table s1**. List of the entries of the Protein Data Bank examined in the present article. |
| --- |
| 1ejg  1gci  1iua  1r6j  1ucs  1us0  1w0n  1x6z  2b97  2ixt  2izq  2ov0  2pve  2vb1  2wfi  3mfj  3ui4  3x2m  3x34  4hp2  4rek  4ua6  5al6  5kwm  5nfm  5tda  5yce  6e6o  6l27  6s2m |

| 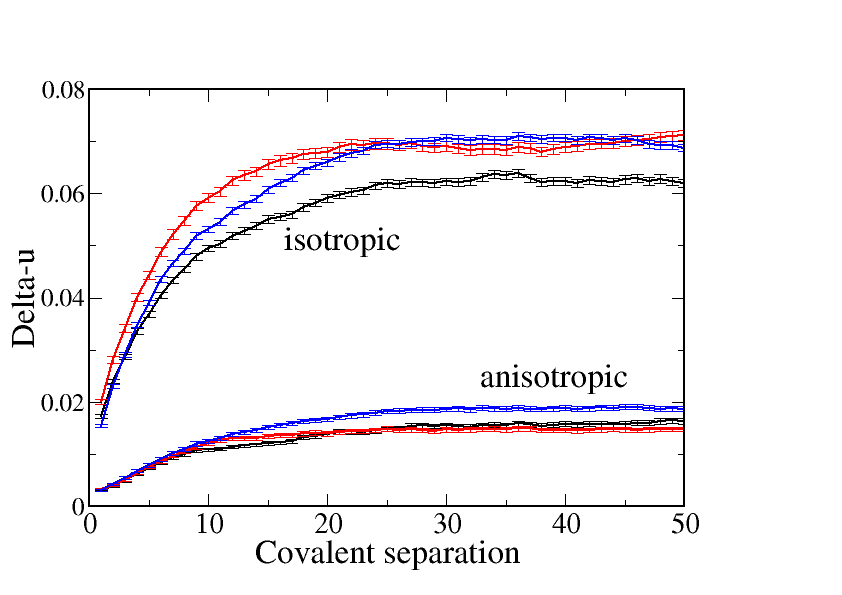 |
| --- |
| **Figure S1**. Relationship between Delta-u and covalent separation in three equally populated subsets of the structures examined in the present communication. |
